# Microneedle radiofrequency for skin rejuvenation: bridging image-derived metrics and photographic assessment

**DOI:** 10.3389/fmed.2025.1710949

**Published:** 2025-11-25

**Authors:** Dajana Malarz, Łukasz Czyżewski, Dorota Olczak-Kowalczyk

**Affiliations:** 1 Department of Geriatric Nursing, Faculty of Health Sciences, Medical University of Warsaw, Warsaw, Poland; 2 Department of Pediatric Dentistry, Faculty of Medicine, Medical University of Warsaw, Warsaw, Poland

**Keywords:** aesthetic medicine, microneedle radiofrequency, wrinkles, adherence, skin analysis

## Abstract

**Purpose:**

To analyze the condition of the skin before and after microneedle radiofrequency (RF) treatment and to identify a series of cascade changes occurring within its structure. Additionally, to test whether regular home care modifies treatment effects and to corroborate objective changes with blinded Global Aesthetic Improvement Scale (GAIS) ratings.

**Materials and methods:**

A retrospective analysis was conducted using photographs of 38 women taken before and after a single microneedle RF treatment and 15 untreated controls. The images were captured using the Observ 520× device at baseline and 30 days post-procedure. Quantitative assessment of changes was performed with ImageJ software. The GAIS was used to evaluate visible changes in the photographs under blinding to group and time. The primary endpoint was the change score (*Δ* = post−pre) for pigmentation, vascular parameters, and hydration across forehead/right/left cheek. Primary analysis used ANCOVA with robust (HC3) errors, adjusting for age and baseline, including home care (regular vs. sporadic/none), a group ×home-care interaction, and false discovery rate (FDR) control.

**Results:**

Among the participants, at least 84.2% showed improvement in skin tone, reduction of pathological erythema, and increased skin hydration. GAIS distributions were bimodal (improvement 1–3 only in RF; no change/worsening 4–5 only in controls; *χ*^2^*p* < 0.001). Unadjusted between-group contrasts favored RF across 9/9 outcomes with large effects. In adjusted models, RF showed an independent benefit for forehead vascular parameters; critically, a significant group × home-care interaction (*p* = 0.003) indicated greater pigmentary gains with regular daily skincare, with hydration showing a similar borderline trend. No serious adverse events were recorded.

**Conclusion:**

Microneedle RF is an effective method for reducing wrinkles and improving image-derived pigmentation, vascular, and hydration metrics at 30 days, with a low complication rate. Regular home care functions as a clinically meaningful response amplifier, particularly for pigmentation- supporting protocolized post-procedure skincare to maximize outcomes. Findings are associative (non-randomized design); replication in randomized, adherence-controlled studies is warranted. Generalizability is limited (women aged 35–50, single center, predominantly Fitzpatrick I–III); extrapolation to men and darker phototypes (IV–VI) is not warranted.

## Introduction

Skin aging is a complex process influenced by both genetic predispositions and environmental factors. Over time, the skin gradually loses its elasticity and firmness, along with a reduction in collagen and elastin fibers. These changes lead to the appearance of wrinkles, hyperpigmentation, dryness, and the loss of the skin’s natural radiance (1).

Contemporary culture, dominated by the pressure to maintain a youthful appearance and focused on aesthetic values, intensifies the desire to combat the signs of aging. A widespread belief that an attractive appearance correlates with professional success, self-confidence, and social acceptance drives an increasing number of people to seek effective and safe methods of skin rejuvenation (2). The growing interest in non-invasive and minimally invasive anti-aging technologies is a direct response to this global trend (3, 4).

Energy-based devices have gained tremendous popularity over the past decades. One of the most innovative methods used in cosmetology and aesthetic medicine is microneedle radiofrequency (RF). A key advantage of this technology is the minimal damage to the epidermis compared to ablative lasers, which results in a much shorter healing and recovery period (5). This technique combines skin needling with the delivery of radiofrequency energy directly into the dermis. During the procedure, microneedles penetrate the skin to a depth of 0.5 to 4.5 mm, depending on the treated area and therapeutic goal. RF energy is delivered through the needles, and the resulting controlled thermal injury stimulates fibroblast activity, neocollagenesis, neoelastogenesis, and remodeling of the extracellular matrix (6, 7). Importantly, this procedure has demonstrated high efficacy in treating wrinkles, acne scars, enlarged pores, loss of firmness, as well as in the reduction of hyperpigmentation and melasma (8).

## Purpose

The aim of this study was to quantify 30-day changes in image-derived measures of pigmentation, vascularity, hydration and the Global Aesthetic Improvement Scale (GAIS) after a single microneedle RF treatment, compared with an untreated control group, and to explore whether regular home care modifies these effects.

## Materials and methods

A retrospective analysis was conducted using photographs of 38 women taken before and after a microneedle RF treatment and 15 women who did not undergo any procedure during the same period (control group). Skin analyzer images were captured on the day of the procedure and again 30 days afterward. Inclusion criteria were: good general health, age between 35 and 50 years with visible signs of skin aging, primarily mimic and static wrinkles, were included in the study, reflecting the most common indication for RF procedures and undergoing a single microneedle RF treatment. Exclusion criteria included: pregnancy, breastfeeding, cancer, age below 35 or above 50, the use of antibiotics or anti-inflammatory medications, autoimmune diseases, and participation in other cosmetic or aesthetic medicine procedures during the study period. Additionally, images of women who did not provide informed consent were excluded from the analysis. These criteria ensured a homogeneous study population that accurately represented typical patients seeking rejuvenation treatments. The selection of participants aged 35–50 years, exclusively women from a single center, was deliberate and controlled. The aim was to minimize the influence of biological variables (e.g., hormone levels, skin thickness) and environmental factors (such as climatic conditions and skincare standards). All photographs were acquired with fixed geometry, maintaining the same camera-to-subject distance and angle and with lighting and exposure settings locked across visits. Participants were predominantly of lighter skin phototypes (Fitzpatrick I–III); darker phototypes (IV–VI) were under-represented in this cohort.

Sample size calculation: 95% confidence level; maximum margin of error ±13.5 percentage points (for proportions under the conservative assumption *p* = 0.50, two sided). Detectable effect: with *N* = 53 (RF *n* = 38, control *n* = 15), the minimum detectable standardised mean difference is Cohen’s d = 0.85 for an unadjusted two group comparison at *α* = 0.05 and 80% power.

Photographs were taken using the Observ 520× device, which utilizes advanced skin imaging technology to assess skin condition across various levels. By applying multiple light filters, the device enabled precise evaluation of pigmentation changes, hydration levels, vascular conditions, and the presence of wrinkles.

The study was conducted in accordance with the Declaration of Helsinki and was approved by the Ethics Committee of the Medical University of Warsaw (AKBE/333/2023, December 11, 2023).

Mathematical measurements of visible changes in the images were performed using ImageJ software. The choice of ImageJ software for quantitative skin image analysis was a deliberate and methodologically justified decision. This tool is widely used in biomedical research, with documented applications in the evaluation of parameters such as pigmentation, vascularity, and skin texture (9). It enables reproducible, mathematically defined measurements and precise delineation of Regions of Interest (ROI), ensuring high repeatability of analyses. ROIs were pre-specified on the forehead and cheeks with systematic exclusion of artifacts (specular highlights, hair, tattoos, pen marks), and the same ROI templates were saved in the ROI Manager and reapplied at the 30-day visit to minimise operator drift. Pigmentation was measured based on the mean gray value within defined ROI covering the forehead and cheeks. Vascularity was determined through analysis of the red channel intensity after prior color histogram normalization, while hydration was assessed indirectly based on the uniformity of light scattering (Light Scattering Uniformity). Operationally, the hydration surrogate was computed as an ROI-level intensity-uniformity index (inverse gray-level variance after input standardisation), interpreted exploratorily as higher uniformity - greater hydration. Notably, this image-derived hydration index has not yet been cross-validated against established biophysical measures (e.g., corneometry, transepidermal water loss); in this study it is treated as an exploratory surrogate. The GAIS was used to evaluate changes observed in the post-treatment photographs and was coded ordinally in ascending order (1 = very much improved, 2 = much improved, 3 = improved, 4 = no change, 5 = worse; lower values indicate better outcomes). Regarding the GAIS scale, despite its subjective nature, full blinding of evaluators to the timing of photographs and study groups (RF vs. control) was implemented, effectively minimizing the risk of cognitive and expectation bias. No validated minimal clinically important difference (MCID) has been established for GAIS in this context, and we did not prespecify responder thresholds; standardized effect sizes are reported for comparability but are not a substitute for validated clinical thresholds. Home care was captured as a single binary, self-reported item (regular = 1 vs. sporadic/none = 0) obtained via a standardized questionnaire, without external validation, diaries, or timestamped product logs. As such, the measure is susceptible to recall and social-desirability biases, and binarization entails loss of intensity information (e.g., product class, frequency, and timing relative to imaging).

IRR was not assessed in this cohort (e.g., via intraclass correlation coefficients). All image analyses were performed by a single trained analyst under a standard operating procedure and with raters blinded to group/time; duplicate reads were not obtained. As such, neither inter- nor intra-rater agreement could be estimated in this cohort. Adverse events (AEs) were captured pragmatically as expected, procedure-related, transient reactions (erythema, edema, and warmth/tenderness). Ascertainment relied on unsolicited reports during/after the session and a structured query at the 30-day visit; no interim diaries, standardized severity grading, or adjudication were implemented, and serious AEs were to be reported immediately if encountered.

### Primary endpoint

For each domain (pigmentation, vascular, and hydration) and facial region (forehead, right cheek, and left cheek), the primary endpoint was the change score defined as *Δ* = post−pre (positive values indicate improvement).

### Primary model

For each metric and region, we fitted an analysis of covariance (ANCOVA) with heteroscedasticity-robust (HC3) standard errors: *Δ* ~ group (RF vs. control) + home care (regular = 1, sporadic/none = 0) + age + baseline value + group × home care. We report regression coefficients (*β*), 95% confidence intervals, and *p*-values; multiple testing was controlled using the Benjamini–Hochberg false discovery rate (FDR) within three families of hypotheses (pigmentation / vascular / hydration).

### Exploratory analyses

(1) Adjusted marginal predictions (means) for RF and control at home-care levels 0/1 (age and baseline held at sample means). (2) Within-group paired t-tests on *Δ* with 95% CIs and Cohen’s d for paired data. (3) Between-group Welch tests on Δ with 95% CIs and Hedges g. (4) Spearman correlations between home care and Δ within each group.

### Statistical analysis

The results underwent descriptive and inferential analyses in SPSS. A two-sided significance level of *α* = 0.05 was adopted. Within-group pre–post comparisons were assessed with paired Student’s *t*-tests; between-group differences in change were evaluated with Welch’s *t*-tests on *Δ* defined as post−pre. Spearman’s rank correlation summarized associations between quantitative/ordinal variables. Group differences in GAIS distributions were examined using the chi-square test. To adjust for covariates, changes (*Δ*) were analysed using ANCOVA (Δ ~ group + baseline + age + home care + group × home care) with HC3 standard errors. The lack of randomization remains a fundamental limitation; accordingly, we interpret the findings as adjusted associations rather than causal effects. Guided by a simplified causal framework (DAG), we identified potential residual confounders, notably motivation/adherence capacity (healthy- adherer effect), socioeconomic status, and patterns of photoprotection/UV exposure. To limit their impact, we included home care and the group × home-care interaction in the models and standardized image acquisition/ROI handling (fixed geometry, locked lighting/exposure, predefined reusable masks). Given the binary, self-reported nature of home care, nondifferential misclassification would be expected to attenuate both main and, in particular, interaction effects (bias toward the null). If misclassification were differential by group, the direction of bias would be unpredictable, warranting cautious interpretation of the group × home-care term. Accordingly, estimates for the group × home-care interaction should be interpreted with particular caution. We emphasize that, despite these steps, residual confounding may persist and cannot be fully eliminated without randomization. For families of related outcomes (pigmentation, vascularity, hydration across three facial regions), *p*-values were adjusted using the Benjamini–Hochberg FDR procedure across all interaction tests. Given the total sample (*N* = 53; RF 38, control 15), the study was powered to detect only large standardized mean differences; interaction contrasts were particularly underpowered. Accordingly, we emphasize effect sizes with 95% CIs over sole reliance on *p*-values, and treat nominal signals that do not survive FDR as hypothesis-generating. Because neither GAIS ratings nor ROI placement/ImageJ extraction were double-read, inter- and intra-rater reliability could not be quantified; consequently, variance estimates may be sensitive to unmeasured rater effects and reproducibility warrants caution. Assumptions for parametric tests (normality of paired differences) were checked; missing data were handled listwise. The study was conducted between May and July 2024.

## Results

Photographs of 38 women taken before and 1 month after microneedle RF treatment were analyzed, along with images of 15 women who did not undergo any treatment during the same time period. The average age was 45 years (RF 44.95 ± 6.58; control 45.20 ± 6.57). Daily skincare routines were reported by 56.6% of the women. Baseline characteristics (age and image-derived metrics across regions) were comparable between groups (see Table 1), and skincare regularity was more common in RF than in controls. No serious adverse events were observed. Expected transient reactions within 24 h included mild erythema and edema at the treatment site; no infections, scarring, or prolonged pain were reported. Given the pragmatic ascertainment window (unsolicited reports and a single 30-day query), under-ascertainment of brief, self-limited reactions between visits cannot be excluded.

**Table 1:** Baseline characteristics (mean ± SD; counts).

Variable	RF (*n* = 38)	Control (*n* = 15)
Age (years)	44.95 ± 6.58	45.20 ± 6.57
Pigmentation—forehead (pre)	170.01 ± 7.66	170.91 ± 12.59
Pigmentation—right cheek (pre)	166.84 ± 7.77	167.08 ± 11.35
Pigmentation—left cheek (pre)	166.39 ± 6.62	164.77 ± 10.31
Vascularity—forehead (pre)	190.70 ± 8.13	187.27 ± 11.34
Vascularity—right cheek (pre)	180.92 ± 7.68	179.66 ± 10.36
Vascularity—left cheek (pre)	184.16 ± 8.64	178.22 ± 11.55
Hydration—forehead (pre)	157.65 ± 4.25	154.77 ± 6.65
Hydration—right cheek (pre)	148.36 ± 4.23	152.71 ± 4.20
Hydration—left cheek (pre)	149.26 ± 3.88	151.93 ± 3.69
Home care: regular/sporadic/none	24/8/6	6/5/4

All photographs from both the treatment and control groups were evaluated using the GAIS scale to determine the degree of improvement or deterioration in skin condition after the procedure (Table 2). A significant improvement was noted in 34.2% of women in the treatment group (*p* = 0.011). In the control group, 60% experienced a deterioration in skin condition, while 40% showed no change. The chi-square test indicated statistically significant differences in the overall improvement level between the treatment and control groups [Chi-square (4) = 43.14; *p* < 0.001]. GAIS ratings showed a strongly bimodal pattern: scores 1–3 occurred only in the RF group; score 5 only in controls; score 4 (no change) appeared sporadically in both groups [*χ*^2^ (4) = 43.14; *p* < 0.001].

**Table 2:** GAIS Scale.

Variable		Group	
		Study	Control
GAIS	Substantial improvement	13 (34.2%)	0 (0%)
	Significant improvement	13 (34.2%)	0 (0%)
	Improvement	9 (23.7%)	0 (0%)
	No change	3 (7.9%)	6 (40%)
	Worsening	0 (0%)	9 (60%)

Based on the mathematical parameters obtained using the ImageJ software, the vascularity, hydration level, and skin tone (including sun-related or hormonal hyperpigmentation) were compared between the treatment and control groups (Table 3). The paired *t*-test revealed that the treatment group showed a statistically significant improvement in nearly all analyzed variables, with the exception of vascularity on the left cheek (*p* = 0.151). In contrast, the paired *t*-test for the control group indicated a statistically significant deterioration in all measured variables (e.g., right-cheek vascularity *p* < 0.001; left-cheek vascularity *p* < 0.001). Between-group comparisons of change favored RF across 9/9 outcomes, with large standardized differences; for example, forehead vascularity difference in change = +3.18 (95% CI 2.16–4.19; *p* < 0.001), right-cheek pigmentation +5.15 (3.15–7.14; *p* < 0.001), and forehead hydration +5.62 (3.28–7.97; *p* < 0.001). Across all nine outcomes, *p* values were < 0.001.

**Table 3:** Comparison of vascularity, hydration level, and skin tone.

Group		Average	Standard deviation	Mean difference	Standard deviation of the differences	*t*	*p*
Study	Pigmentation—forehead before	170.01	7.66	−1.94	3.86	*t*(37) = −3.10	0.004
	Pigmentation—forehead after	171.96	6.98				
	Pigmentation—right cheek before	166.84	7.77	−2.76	4.97	*t*(37) = −3.42	0.002
	Pigmentation—right cheek after	169.60	8.53				
	Pigmentation—left cheek before	166.39	6.62	−2.05	3.39	*t*(37) = −3.72	<0.001
	Pigmentation—left cheek after	168.44	6.38				
	Vascularity—forehead before	190.70	8.13	−1.76	2.22	*t*(37) = −4.90	<0.001
	Vascularity—forehead after	192.47	7.51				
	Vascularity—right cheek before	180.92	7.68	−1.66	2.97	*t*(37) = −3.44	0.001
	Vascularity—right cheek after	182.58	7.36				
	Vascularity—left cheek before	184.16	8.64	−1.01	4.27	*t*(37) = −1.47	0.151
	Vascularity—left cheek after	185.17	8.76				
	Hydration—forehead before	157.65	4.25	−2.69	3.20	*t*(37) = −5.18	<0.001
	Hydration—forehead after	160.34	4.64				
	Hydration—right cheek before	148.36	4.23	−2.94	3.71	*t*(37) = −4.89	<0.001
	Hydration—right cheek after	151.30	5.23				
	Hydration—left cheek before	149.26	3.88	−1.62	2.90	*t*(37) = −3.45	0.001
	Hydration—left cheek after	150.88	4.84				
Control	Pigmentation—forehead before	170.91	12.59	1.85	2.14	*t*(14) = 3.36	0.005
	Pigmentation—forehead after	169.06	13.97				
	Pigmentation—right cheek before	167.08	11.35	2.39	2.24	*t*(14) = 4.12	0.001
	Pigmentation—right cheek after	164.69	11.05				
	Pigmentation—left cheek before	164.77	10.31	1.52	1.99	*t*(14) = 2.95	0.010
	Pigmentation—left cheek after	163.25	10.14				
	Vascularity—forehead before	187.27	11.34	1.41	1.37	*t*(14) = 4.00	0.001
	Vascularity—forehead after	185.86	11.48				
	Vascularity—right cheek before	179.66	10.36	1.04	0.86	*t*(14) = 4.68	<0.001
	Vascularity—right cheek after	178.63	10.38				
	Vascularity—left cheek before	178.22	11.55	2.35	2.02	*t*(14) = 4.52	<0.001
	Vascularity—left cheek after	175.87	11.36				
	Hydration—forehead before	154.77	6.65	2.93	3.89	*t*(14) = 2.92	0.011
	Hydration—forehead after	151.83	5.57				
	Hydration—right cheek before	152.71	4.20	1.32	1.53	*t*(14) = 3.32	0.005
	Hydration—right cheek after	151.39	5.03				
	Hydration—left cheek before	151.93	3.69	1.55	1.54	*t*(14) = 3.91	0.002
	Hydration—left cheek after	150.37	3.55				

In the treatment group, improvement in skin tone, analyzed separately for specific facial areas, was observed in at least 84.2% of participants. A reduction in pathological erythema was also noted in 84.2% of women. An increased level of skin hydration was documented in at least 84.2% of the subjects (Table 4). Within-group analyses mirrored these patterns: RF improved 8/9 outcomes (non-significant only for left-cheek vascularity, change = +1.01; 95% CI −0.39 to 2.42; *p* = 0.151), whereas controls worsened in 9/9 outcomes (e.g., forehead hydration change = − 2.93; 95% CI −5.08 to −0.78; *p* = 0.011).

**Table 4:** Changes in skin tone, vascularity, and hydration.

Variable		Group	
		Study	Control
Pigmentation—forehead	Worsening	5 (13.2%)	14 (93.3%)
	No change	1 (2.6%)	1 (6.7%)
	Improvement	32 (84.2%)	0 (0%)
Pigmentation—right cheek	Worsening	5 (13.2%)	14 (93.3%)
	No change	0 (0%)	0 (0%)
	Improvement	33 (86.8%)	1 (6.7%)
Pigmentation—left cheek	Worsening	4 (10.5%)	12 (80%)
	No change	0 (0%)	1 (6.7%)
	Improvement	34 (89.5%)	2 (13.3%)
Vascularity—forehead	Worsening	2 (5.3%)	13 (86.7%)
	No change	0 (0%)	0 (0%)
	Improvement	36 (94.7%)	2 (13.3%)
Vascularity—right cheek	Worsening	5 (13.2%)	14 (93.3%)
	Improvement	33 (86.8%)	1 (6.7%)
Vascularity—left cheek	Worsening	6 (15.8%)	14 (93.3%)
	No change	0 (0%)	0 (0%)
	Improvement	32 (84.2%)	1 (6.7%)
Hydration—forehead	Worsening	4 (10.5%)	15 (100%)
	Improvement	34 (89.5%)	0 (0%)
Hydration—right cheek	Worsening	6 (15.8%)	13 (86.7%)
	Improvement	32 (84.2%)	2 (13.3%)
Hydration—left cheek	Worsening	6 (15.8%)	15 (100%)
	Improvement	32 (84.2%)	0 (0%)

Table 5 presents data obtained through Spearman’s rank correlation analysis, which showed that in the treatment group, there was no statistically significant correlation between age and any of the studied variables. However, a significant correlation was found between the regularity of daily skincare routines and GAIS scores (rho = −0.742; *p* < 0.001) – the more consistent the skincare, the greater the improvement observed, pigmentation improvement – regular skincare was associated with more noticeable improvements in discoloration, hydration levels – more regular daily care correlated with increased skin hydration. In the control group, Spearman’s rank correlation analysis showed no statistically significant relationships between age and any of the variables, nor between the consistency of daily skincare and any of the studied outcomes. These findings support the interpretation that regular home care amplifies the benefits after RF rather than acting independently.

**Table 5:** Spearman correlations between skin improvement and age as well as daily skincare routine.

Variable		Age	Skin routine
GAIS	Correlation coefficient	0.182	−0.742
	*p*	0.273	<0.001
Pigmentation—forehead	Correlation coefficient	−0.144	0.375
	*p*	0.389	0.020
Pigmentation—right cheek	Correlation coefficient	−0.111	0.732
	*p*	0.508	<0.001
Pigmentation—left cheek	Correlation coefficient	0.042	0.339
	*p*	0.801	0.038
Vascularity—forehead	Correlation coefficient	0.044	0.141
	*p*	0.791	0.398
Vascularity—right cheek	Correlation coefficient	0.149	0.201
	*p*	0.372	0.227
Vascularity—left cheek	Correlation coefficient	0.118	0.154
	*p*	0.479	0.355
Hydration—forehead	Correlation coefficient	−0.097	0.538
	*p*	0.562	<0.001
Hydration—right cheek	Correlation coefficient	−0.047	0.518
	*p*	0.777	<0.001
Hydration—left cheek	Correlation coefficient	−0.099	0.593
	*p*	0.555	<0.001

In adjusted models (ANCOVA with HC3), the RF group retained a significant advantage for forehead vascularity (*β* = 3.28; 95% CI 1.66–4.91; *q* = 0.0002, *p* < 0.001), while other regions showed positive but non-significant effects after FDR (Table 6). A significant group × home-care interaction was observed for right-cheek pigmentation (*β* interaction = 5.45; 95% CI 1.72–9.18; *q* = 0.030), indicating that regular home care amplified the RF effect; for cheek hydration, interactions were nominal (*p* < 0.05) but did not survive FDR (*q* = 0.103 right; *q* = 0.077 left) (Table 7). These adjusted findings align with the unadjusted contrasts and support the clinical relevance of combining RF with regular home care.

**Table 6:** Spearman correlations between skin improvement and age as well as daily skincare routine.

Variable		Age	Skin routine
GAIS	Correlation coefficient	0.182	−0.742
	*p*	0.273	<0.001
Pigmentation—forehead	Correlation coefficient	−0.144	0.375
	*p*	0.389	0.020
Pigmentation—right cheek	Correlation coefficient	−0.111	0.732
	*p*	0.508	<0.001
Pigmentation—left cheek	Correlation coefficient	0.042	0.339
	*p*	0.801	0.038
Vascularity—forehead	Correlation coefficient	0.044	0.141
	*p*	0.791	0.398
Vascularity—right cheek	Correlation coefficient	0.149	0.201
	*p*	0.372	0.227
Vascularity—left cheek	Correlation coefficient	0.118	0.154
	*p*	0.479	0.355
Hydration—forehead	Correlation coefficient	−0.097	0.538
	*p*	0.562	<0.001
Hydration—right cheek	Correlation coefficient	−0.047	0.518
	*p*	0.777	<0.001
Hydration—left cheek	Correlation coefficient	−0.099	0.593
	*p*	0.555	<0.001

**Table 7:** Group × home-care interaction (*β*_interaction, *p*, FDR).

Metric (region)	*β* interaction (95% CI)	*p*	FDR
Pigmentation—right cheek	5.45 (1.72, 9.18)	0.003	0.030
Hydration—right cheek	3.58 (0.18, 6.98)	0.034	0.103
Hydration—left cheek	2.81 (0.44, 5.18)	0.017	0.077
Others	0.11–2.81	0.196–0.922	>0.29

Others include: vascularity (forehead/right/left), pigmentation (forehead/left cheek), hydration (forehead), Δ = post−pre (higher = greater improvement). Model: ANCOVA adjusted for baseline value and age; robust standard errors (HC3). Two-sided *p*-values. Multiplicity controlled with Benjamini–Hochberg FDR across all interaction tests in this table; significance defined as *q* < 0.05. A positive *β* interaction indicates a larger home-care–related gain in the RF group than in controls. FDR, false discovery rate.

## Discussion

In recent years, microneedle RF has gained significant importance in aesthetic dermatology and cosmetology as a modern, minimally invasive method used for skin rejuvenation, wrinkle reduction, and the treatment of acne scars. This technology combines the effects of controlled mechanical micro-injury with the selective heating of tissues using high-frequency current, enabling the stimulation of intensive regenerative processes without significant disruption to the skin surface.

In baseline-adjusted ANCOVA the most robust main effect was observed for forehead vascularity (*β* = 3.28, 95% CI 1.66–4.91, *p* < 0.001; Table 6). Effects at other sites were directionally favorable to RF but statistically inconclusive after adjustment and multiplicity. For prespecified moderation by home-care, only the pigmentation interaction on the right cheek survived false-discovery control (*β*_interaction = 5.45, 95% CI 1.72–9.18, *p* = 0.003, *q* = 0.030; Table 7). Hydration interactions on the cheeks were nominally significant before multiplicity (right cheek *β* interaction = 3.58, *p* = 0.034, *q* = 0.103; left cheek *β*_interaction = 2.81, *p* = 0.017, *q* = 0.077) but did not meet the FDR threshold, which, given the modest sample and group imbalance (38 vs. 15), raises the possibility of both Type I (false positives) and Type II (false negatives). The “left-cheek vascularity” signal warrants comment: the adjusted group effect was positive but non-significant (*β* = 2.32, 95% CI −0.67 to 5.31, *p* = 0.128; Table 6), and no vascular interaction survived FDR (Table 7). Accordingly, signals that did not pass FDR- including the cheek-level hydration interactions and the left-cheek vascularity contrast- should be regarded as hypothesis-generating rather than confirmatory. This pattern argues against a reproducible biological anomaly and is more parsimoniously explained by lateral anatomical and environmental asymmetry (for example habitual sun exposure or sleep side) together with variance limitations at cheek sites; however, we did not capture side dominance, sleep position, or laterality of sun exposure, so this explanation remains provisional. Prospective work will address this with side-balanced ROI templates, explicit laterality covariates, and adequate powering for interaction contrasts.

Despite statistical adjustment, the absence of randomization remains a key limitation for causal inference; accordingly, we interpret the effects as adjusted associations. The observed differences in health-related behaviors (more frequent regular home care in the RF group- Table 1) likely correlate with motivation, socioeconomic status, and photoprotection habits, which could inflate the apparent RF effect independently of the intervention per se. Less commonly, attenuation is also possible (e.g., regression to the mean in the context of poorer baseline status in the RF group). The lack of randomization is a fundamental constraint that cannot be fully compensated by any statistical correction.

Including home care and the group × home-care interaction mitigates bias from measured sources, yet unmeasured components (e.g., true adherence, phototype-specific differences, and under-captured environmental exposures) may still shift estimators in either direction. Moreover, the self-reported, binary operationalization of home care increases the risk of misclassification, which could attenuate or spuriously amplify the interaction. The absence of IRR assessment for GAIS and ROI segmentation constitutes an additional source of uncertainty.

In light of these considerations, we interpret the main RF-versus-control effects conservatively, and we view the RF + home-care synergy as a hypothesis supported by concordant directional evidence (nonparametric analyses, blinded GAIS) that nonetheless requires confirmation in randomized trials with standardized monitoring of the skincare protocol. While GAIS is a widely used, clinically recognizable instrument for global aesthetic improvement in real-world studies, it lacks a validated MCID in this context and we did not quantify inter-rater reliability in this cohort. In retrospective designs based on archived images, histology is not feasible; accordingly, future prospective work should prioritize non-invasive validation (e.g., high-frequency ultrasound, OCT, 3D profilometry/texture, objective colorimetry) and, where feasible, minimally invasive biomarker approaches (e.g., tape-strip assays).

Image-derived hydration and tone indices in this study are exploratory surrogates without an established minimal clinically important difference; accordingly, we interpret their 30-day changes primarily through standardized effects and concordance with blinded GAIS, rather than absolute unit thresholds. We will prospectively report Cohen’s d (or percent change from baseline) with 95% CIs, and define responder cut-offs against external anchors in the extended 3–6-month follow-up.

Beyond the present red- channel with histogram normalisation approach, the literature describes erythema quantification from the a* axis in the CIE L*a*b* space, which has demonstrated high inter-observer agreement and treatment responsiveness in rosacea cohorts (9). Within that framework, a commonly used contrast metric is *Δ* a*, defined as a* (lesional ROI)–a*(reference non-lesional skin, e.g., neck), and can be adopted by external groups seeking alignment with published ImageJ workflows (9, 10). In laboratories employing physical color targets, logging the green-channel intensity of a calibration sticker can help standardise R/G across sessions; this step is optional and primarily relevant to cross-session harmonisation rather than within-session comparisons (9, 10). Our study prioritised the red-channel + histogram-normalisation pipeline under tightly standardised acquisition (fixed geometry, locked lighting/exposure) to maximise within-cohort reproducibility, while presenting the CIELAB/ *Δ* a* pathway as methodological context to facilitate replication in settings where that standard is preferred (9, 10).

Cross-platform considerations. Because there is no single non-invasive gold standard, and cross-device studies document partial non-interchangeability among commercial systems (e.g., VISIA vs. Antera 3D/CSKIN; OBSERV 520× vs. Dermacatch vs. VISIA), cross-pipeline agreement cannot be assumed *a priori* (11–13). Accordingly, results should be interpreted with awareness of these platform effects, and external implementations are encouraged to conduct local color/geometry checks and, where feasible, head-to-head calibration before pooling data across devices (11–13). Prospective work will directly compare the red-channel and CIELAB/Δa* pipelines in our setting and report cross-pipeline agreement to support broader harmonisation (9–13).

Microneedle RF has been widely applied in skin rejuvenation and the treatment of selected dermatological conditions, and its use has already been extensively reported in the literature. However, previous studies have predominantly focused on specific aspects of efficacy, such as wrinkle reduction or skin firmness improvement. In the present study, a more comprehensive approach was adopted, encompassing the simultaneous assessment of several key skin parameters: wrinkles, pigmentation, vascular changes, erythema, hydration, and overall skin tone uniformity. Such a broad evaluation made it possible to capture the complex and multidirectional effects of the therapy, more accurately reflecting the actual needs of patients undergoing aesthetic procedures. An innovative element of this study was the inclusion of home skin care as an important factor influencing the effectiveness of RF. To date, only a few studies have examined the synergy between professional treatment and daily care routines, despite clear clinical evidence of their importance in maintaining and enhancing therapeutic outcomes. Another distinguishing feature of this research was its implementation under real clinical conditions, in a group of patients presenting with typical aesthetic concerns. As a result, the findings are not only scientifically relevant but also directly applicable to everyday clinical practice for physicians and cosmetologists. The results confirmed the high efficacy of RF in improving skin quality, as well as the safety of the procedure, since only mild and transient adverse effects were observed. This integrated approach, combining multi-parameter skin evaluation, home care, and real-life clinical context, represents a novel contribution and highlights the added value of the present work compared to existing publications.

Unlike laser-based technologies, radiofrequency efficacy is not chromophore-dependent in principle. However, our cohort comprised predominantly Fitzpatrick I–III; therefore, our data do not directly inform response or safety in IV–VI, and any extrapolation to darker phototypes should be made with caution. The dynamic development of devices- from bipolar systems to fractional microneedle applicators—has enabled precise treatment at different dermal depths while ensuring a short recovery time and minimal risk of side effects. The safety of the procedure was an important aspect of evaluating RF efficacy. Throughout the entire observation period, no serious or long-term adverse effects related to the therapy were reported. The most common side effects were transient redness, swelling, and a warm sensation at the treatment site, all of which resolved spontaneously within 24 h. No cases of infection, keloid formation, or chronic pain were observed. These findings are consistent with literature reports indicating that microneedle RF has a favorable safety profile.

The study results indicated that patients in the RF treatment group achieved greater improvement in skin pigmentation and hydration, while also more frequently reporting regular use of home skin care routines. This discrepancy may have several explanations. First, the very fact of participating in the study and receiving an intervention could have increased patients’ engagement in daily skin care, representing an example of an “active participation effect.” Second, experiencing visible improvement after the procedure may have motivated participants to maintain and enhance the outcomes through consistent care, leading to a positive feedback loop. Third, it should be considered that the RF procedure itself, through microneedling and stimulation of skin regeneration, may increase the bioavailability of active ingredients contained in topical cosmetic products, thereby naturally amplifying the effects of home care. It is important to emphasize that these phenomena do not diminish the therapeutic value of RF, but rather highlight its synergistic nature when combined with appropriate skin care practices. This perspective carries particular practical significance, as it demonstrates that the efficacy of the treatment should not be evaluated in isolation from patients’ behavioral patterns. The findings of this study suggest that the integration of professional therapy with daily home care is a key factor in determining long-term improvements in skin quality. Our adjusted models support this interpretation: the group × home-care interaction for pigmentary outcomes on the right cheek was significant (*β* interaction = 5.45, 95% CI 1.72–9.18, *p* = 0.003), indicating that regular, protocolised skincare acts as a response amplifier after RF rather than a stand-alone driver of change. Hydration interactions on the cheeks were nominal before multiplicity (right: *β* interaction = 3.58, 95% CI 0.18–6.98, *p* = 0.034; left: *β* interaction = 2.81, 95% CI 0.44–5.18, *p* = 0.017) and no vascular interaction survived FDR. Consistent with this pattern, the strongest main effect was observed for forehead vascularity (*β* = 3.28, 95% CI 1.66–4.91, *p* < 0.001), whereas vascular changes at other regions were modest and region-dependent. These moderation signals should therefore be regarded as hypothesis-generating given the binary, self-reported exposure and its susceptibility to misclassification, which can materially impact interaction estimates.

Clinical studies confirm that microneedle RF can increase skin thickness by over 40%, improve its firmness, and enhance overall skin revitalization, all with low risk of complications and brief downtime (14). A key advantage of the treatment is the even delivery of heat to the dermis with minimal damage to the epidermis (15–17). Studies by Kim et al. (18, 19) and Kwon et al. (20) confirm wrinkle reduction in the eye area by 25 to 43% after three sessions, with effects lasting up to 6 months. Cho et al. (21) described three cases showing improved wrinkle depth in the eye area. In a 6-month follow-up, 82% of women reported improvement exceeding 25%. Yogya et al. (22) reported a wrinkle reduction of 25%–75% in the eye region, without adverse effects such as infection, erythema, or scarring.

Our own research confirms improved skin elasticity in 92.1% of participants in the treatment group. Studies by Wu et al. also reported significant aesthetic improvement in the eye area treated with microneedle RF, in both patient and physician assessments (23).

Seul et al. (24) investigated the effectiveness and safety of pulsed bipolar radiofrequency in treating acne lesions and post-inflammatory erythema. Most patients showed significant clinical improvement after three treatments at four-week intervals (all *p* < 0.05). Ruiz-Esparza et al. (25) applied microneedle RF in 22 patients with active acne and observed at least 75% reduction in active lesions in 82% of cases.

Liu et al. (26) assessed the effectiveness and safety of fractional microneedle RF in treating facial photoaging. Using a split-face model, one half of the face received treatment while the other served as a control. The treated side showed significant improvements in both clinical and patient self-assessments at 1 and 3 months post-treatment. A marked reduction in skin roughness was also observed on the treated side.

Our findings confirm significant improvements in skin quality parameters in the microneedle RF group (e.g., forehead hydration *p* < 0.001; left-cheek pigmentation *p* < 0.001). Improvement in skin tone, assessed separately for selected facial areas, was observed in at least 84.2% of participants. A reduction in pathological erythema was also noted in 84.2% of women. Notably, an increase in skin hydration was observed in at least 84.2% of the treated group, indicating the multifaceted therapeutic potential of microneedle RF. Another observation concerned the limited improvement in vascular changes on the left cheek within the treatment group, despite noticeable differences on the opposite cheek and forehead (adjusted contrast *β* = 2.32, 95% CI −0.67 to 5.31; *p* = 0.128). This likely reflects the natural asymmetry in the distribution of vascular features across the face and individual variability in skin response, influenced by factors such as epidermal thickness, vascular density, and prior skin damage. While RF can induce remodeling of dermal structures and may indirectly affect microvascular patterns, it was primarily designed to reduce wrinkles and improve overall skin tone rather than to directly diminish erythema or telangiectasias. These findings highlight that, although RF effectively enhances multiple parameters of skin quality, its impact on vascular lesions is limited and may require adjunctive procedures for targeted management. Clinically, this underscores the importance of individualized, multimodal approaches when planning aesthetic interventions.

An analysis by Hongcharu and Gold (27) showed that SmartScan™ Nano-Fractional RF technology effectively improved skin texture and pigmentation. Participants observed a reduction in wrinkles, hyperpigmentation, and acne-related redness. Nilforoushzadeh et al. (28) found significant reduction in pore size and pigmentation spots, a decrease in transepidermal water loss by 18.44%, and an increase in skin density by 44.41%. Ultrasound assessments revealed increased epidermal and dermal thickness and density.

In a study by Peterson et al. (29), the effectiveness of combining fractional laser with radiofrequency versus RF alone was evaluated in acne scar treatment. After five treatments at 30-day intervals, average improvements of 72.3% in scar grading, 68.2% in scar appearance, 66.7% in skin texture, and 13.3% in pigmentation were recorded. Subjective assessments indicated a 60% overall improvement (*p* = 0.02). The study suggests that combining fractional laser with RF may be an effective strategy for acne scar management.

The analysis of the results revealed a particularly important observation that requires discussion. The deterioration of skin condition observed in 60% of participants in the control group may be explained by the ongoing aging process, which can progress dynamically even within a relatively short time frame. This phenomenon may have been further influenced by environmental factors such as UV radiation, air pollution, or a diet low in antioxidants, as well as lifestyle factors including smoking and chronic stress. The absence of any intervention in this group allowed the natural degenerative processes of the skin to proceed unhindered, which explains the noticeable decline in skin quality parameters.

Mechanistically, the domain-specific pattern aligns with RF biology: controlled dermal heating reliably triggers neocollagenesis and matrix remodeling (supporting GAIS and hydration/tone), whereas direct ablation of superficial vascular structures is not a primary RF target. Regular, SPF-anchored home care reduces UV-driven melanogenesis and stabilizes melanocyte activity; retinoids and pigment modulators accelerate epidermal turnover, coherently explaining the stronger pigmentary gains among adherent patients after RF. The limited adjusted change for left-cheek vascularity, despite favorable trends elsewhere, likely reflects anatomical–environmental asymmetry and underscores the non-vascular-targeted nature of RF. However, we did not collect histologic, high-resolution dermal imaging (e.g., reflectance confocal microscopy or optical coherence tomography), or skin-surface biomarker data in this cohort; consequently, mechanistic interpretations (including the putative RF × home-care synergy) should be regarded as biologically plausible but unvalidated in this study.

From a clinical standpoint, RF outcomes should be embedded in a standardized, adherence-dependent home-care pathway (gentle cleanser, daily broad-spectrum photoprotection, nighttime retinoid or alternative for sensitive skin, and a pigment-modulating agent when indicated). For patients with prominent vascular components (erythema/telangiectasias), a multimodal strategy is advisable: vascular-selective light/laser to target chromophore-driven lesions, with RF contributing texture, tone, and collagen remodeling. The deterioration observed in 60% of controls over 30 days underscores the real-world trajectory of untreated skin parameters and supports timely intervention when clinically appropriate.

The 30-day observation period was deliberately adopted in the study design to eliminate potential confounding factors such as patients undergoing additional aesthetic procedures, seasonal environmental variations, or inconsistencies in home skincare routines. Despite the relatively short follow-up, achieving such pronounced clinical and imaging outcomes within this timeframe after a minimally invasive procedure confirms the efficacy and safety of RF treatment. According to current histological evidence, collagen fiber remodeling occurs over 3 to 6 months; therefore, a continued enhancement of results can be expected in the longer term, further supporting the high clinical value of the findings. In addition, the harms assessment was pragmatic rather than systematic; future studies should incorporate standardized adverse-event reporting with predefined solicited reactions, severity grading, and multiple time-points to better characterize safety and durability. Given the 30-day horizon, these results should be regarded as early outcomes and do not establish the durability of collagen remodeling or long-term clinical benefit. Reliance on blinded GAIS and standardized effect sizes improves interpretability but does not compensate for the absence of validated clinical thresholds. Future work will pre-specify anchor-based and distribution-based MCIDs (e.g., against patient global ratings) and include longer follow-up (3–6 months) to evaluate durability.

### Limitations

IRR for both GAIS and the ImageJ/ROI workflow was not quantified, and no duplicate reads were obtained; this limits reproducibility and may affect precision of effect estimates. Although raters were fully blinded to group allocation and time point, we did not compute concordance indices (e.g., ICC or weighted *κ*), and thus cannot provide an empirical estimate of rating precision and stability in this sample. Consequently, interpretation of GAIS-based findings should acknowledge this methodological constraint. Limitations encompass the retrospective, non-randomized design (potential selection and residual confounding), single-center cohort with predominantly lighter phototypes (Fitzpatrick I-III) (generalizability), a 30-day horizon (no durability curve). A further limitation is the non-systematic harms capture (unsolicited reports plus a single 30-day query, without standardized grading), which may under-estimate transient or delayed reactions; findings should be interpreted accordingly, and future work should adopt standardized AE frameworks. Moreover, reliance on GAIS and standardized effect sizes, while informative, cannot substitute for validated, clinically meaningful thresholds, and thus clinical significance should be interpreted cautiously. A further major limitation is the absence of biological validation, no histology, high-resolution dermal imaging, or tape-strip biomarker assays were collected to corroborate collagen remodeling or melanogenesis modulation, so the proposed mechanisms and the RF × home-care moderation remain inferential.

Beyond motivation and socioeconomic status, we identify additional sources of potential residual confounding: real-world photoprotection and UV exposure patterns (including seasonality), smoking, sleep and stress, the composition and timing of the most recent home-care application (retinoids, melanogenesis inhibitors, occlusion), systemic medications, subtle phototype and hormone-related differences, environmental exposures (pollution, humidity/temperature), and measurement factors (make-up removal, color/positioning calibration, absence of IRR), as well as the lack of laterality covariates (sleep side, driving/sun exposure side). Some of these may inflate observed between-group differences (e.g., superior photoprotective habits among more adherent participants; see the higher proportion of regular home care in the RF arm, Table 1), whereas others may attenuate effects (e.g., regression to the mean or greater physical activity with transient vasodilation in the RF group). A key limitation is the binary, self-reported home-care measure, unvalidated and single time point which is vulnerable to recall and social desirability biases; nondifferential error would tend to attenuate moderation, whereas differential error could bias the group × home-care interaction in either direction. Findings should be interpreted accordingly. Accordingly, findings should not be extrapolated to men, other age bands, or darker phototypes (Fitzpatrick IV–VI) without caution; confirmation in broader, multicenter cohorts is warranted, while causal inference remains limited.

The small sample size yields wide confidence intervals and low power, especially for interaction tests; despite Benjamini–Hochberg control, residual Type I error cannot be excluded, and the risk of Type II error is non-trivial.

### Future directions

Prospective randomized studies stratified by pre-specified skincare adherence, standardized adverse event monitoring, and longer follow-up (e.g., 3 or 6 months) are warranted to confirm durability and optimize effect size. Adding IRR for blinded GAIS and harmonizing Observ 520×/ImageJ pipelines will improve reproducibility. Mechanistic sub-studies (barrier function, pigmentation biomarkers) and multimodal arms (RF ± vascular-selective devices) may further personalize patient-level algorithms. We will broaden external generalisability by expanding recruitment to men, wider age bands, and darker Fitzpatrick phototypes (IV–VI) in a multicentre design. Stratified enrolment by sex, age, and phototype will enable formal effect-heterogeneity analyses and improve transportability of findings.

## Conclusion

In this single-center cohort of women aged 35–50 years, predominantly Fitzpatrick I–III, microneedle RF was associated with improvements in wrinkles, pigmentation, vascular parameters, and erythema at 30 days. Microneedle RF treatments lead to visible improvements in skin smoothness, tone uniformity, and overall skin condition, making this an attractive therapeutic option in aesthetic medicine. Furthermore, this method is associated with a low complication rate, making it safe and well-tolerated by patients. This supports its increasing popularity among individuals seeking non-invasive skin enhancement solutions.

Importantly, our adjusted analyses confirmed an independent microneedle RF benefit on forehead vascular parameters. We also found a clear synergy with regular home care: patients who adhered to daily skincare achieved markedly greater pigmentary gains than those with sporadic/no care. Clinically, this supports embedding microneedle RF in a standardized home-care pathway (gentle cleanser, daily broad-spectrum SPF, evening retinoid/alternative, and when indicated a pigment-modulating agent), with explicit adherence counseling. Conversely, limited change for left-cheek vascularity underscores anatomical/environmental asymmetry and that RF is not a vascular-targeted modality; consider adding vascular-selective light/laser for predominant erythema/telangiectasias.

From an innovation standpoint, this real-world, multiparametric study with blinded ratings and a formal interaction test offers pragmatic guidance on operationalizing microneedle RF plus adherence-sensitive home care in routine practice. Future work should prospectively validate these findings in randomized, adherence-stratified designs with systematic safety capture and evaluate multimodal combinations to personalize outcomes. Generalizability is limited (women, 35–50 years, single-center, predominantly Fitzpatrick I–III); broader sex/age/phototype cohorts are needed. Given the absence of randomization and the potential for residual confounding, our conclusions represent adjusted associations and require prospective confirmation in randomized controlled trials.

## Data Availability

The raw data supporting the conclusions of this article will be made available by the authors, without undue reservation.

## References

[1] Makrantonaki E Zouboulis CC. Molecular mechanisms of skin aging: state of the art. Drug Discov Today Dis Mech. (2007) 4:e243–9. doi: 10.1016/j.ddmec.2007.05.002 18056953

[2] Fisher GJ Varani J Voorhees JJ. Looking older: fibroblast collapse and therapeutic implications. J Invest Dermatol. (2002) 119:1059–64. doi: 10.1046/j.1523-1747.2002.19531.x, PMID: 18490597 PMC2887041

[3] Jović M Sforza M Jovanović M Jović M. The acceptance of cosmetic surgery scale: confirmatory factor analyses and validation among Serbian adults. Curr Psychol. (2017) 36:707–18. doi: 10.1007/s12144-016-9458-7, PMID: 29200800 PMC5696501

[4] Hotta TA. Plastic surgical and nonsurgical procedure statistics 2014. Plast Surg Nurs. (2015) 35:53–4. doi: 10.1097/PSN.0000000000000089, PMID: 26020467

[5] Chandra S Mysore V Shah S Malayanur D Shivani SR. Physics of fractional microneedle radiofrequency - a review. J Cutan Aesthet Surg. (2024) 17:177–83. doi: 10.25259/jcas_98_23, PMID: 39483649 PMC11497551

[6] Lee KR Lee EG Lee HJ Yoon MS. Assessment of treatment efficacy and sebosuppressive effect of fractional radiofrequency microneedle on acne vulgaris. Lasers Surg Med. (2015) 47:559–65. doi: 10.1002/lsm.22388, PMID: 24249246

[7] Hantash BM Ubeid AA Chang H Kafi R Renton B. Bipolar fractional radiofrequency treatment induces neoelastogenesis and neocollagenesis. Lasers Surg Med. (2009) 41:1–9. doi: 10.1002/lsm.20731, PMID: 19143021

[8] Gold MH. Microneedling with radiofrequency for acne scars in Asian skin: a review and update. J Cosmet Dermatol. (2014) 13:212–23. doi: 10.1111/jocd.12100, PMID: 25196689

[9] Logger JGM de Jong EMGJ Driessen RJB van Erp PEJ. Evaluation of a simple image-based tool to quantify facial erythema in rosacea during treatment. Skin Res Technol. (2020) 26:804–12. doi: 10.1111/srt.12878, PMID: 32537843 PMC7754330

[10] Tao M Li M Zhang Y Liu Y Jiang P Liu Y . Objectively quantifying facial erythema in rosacea aided by the ImageJ analysis of VISIA red images. Skin Res Technol. (2023) 29:e13241. doi: 10.1111/srt.13241, PMID: 36426837 PMC9838746

[11] Wang X Shu X Li Z Huo W Zou L Tang Y . Comparison of two kinds of skin imaging analysis software: VISIA® from canfield and IPP® from media cybernetics. Skin Res Technol. (2018) 24:379–85. doi: 10.1111/srt.12440, PMID: 29377397

[12] Zuo Y Li A He H Wan R Li Y Li L. Assessment of features in facial hyperpigmentation: comparison study between VISIA and CSKIN. Skin Res Technol. (2022) 28:846–50. doi: 10.1111/srt.13216, PMID: 36308512 PMC9907609

[13] Huang YW Arkesteijn W Lai YJ Ng CY. A comparative study of an advanced skin imaging system in diagnosing facial pigmentary and inflammatory conditions. Sci Rep. (2024) 14:14673. doi: 10.1038/s41598-024-63274-7, PMID: 38918427 PMC11199608

[14] Nilforoushzadeh MA Heidari-Kharaji M Shahverdi M Nouri M Enamzadeh R Nobari NN . Microneedle fractional radiofrequency in the treatment of periorbital dark circles. J Cosmet Dermatol. (2023) 22:2218–24. doi: 10.1111/jocd.15870, PMID: 37326254

[15] Shin JM Kim JE. Radiofrequency in clinical dermatology. Med Lasers. (2013) 2:49–57. doi: 10.25289/ML.2013.2.2.49

[16] Elsaie ML Choudhary S Leiva A Nouri K. Nonablative radiofrequency for skin rejuvenation. Dermatologic Surg. (2010) 36:577–89. doi: 10.1111/j.1524-4725.2010.01510.x, PMID: 20384760

[17] Tan MG Jo CE Chapas A Khetarpal S Dover JS. Radiofrequency microneedling: a comprehensive and critical review. Dermatologic Surg. (2021) 47:755–61. doi: 10.1097/DSS.0000000000002972, PMID: 33577211

[18] Lee SJ Kim JI Yang YJ Nam JH Kim WS. Treatment of periorbital wrinkles with a novel fractional radiofrequency microneedle system in dark-skinned patients. Dermatologic Surg. (2015) 41:615–22. doi: 10.1097/DSS.0000000000000216, PMID: 25899885

[19] Kim JK Roh MR Park GH Kim YJ Jeon IK Chang SE. Fractionated microneedle radiofrequency for the treatment of periorbital wrinkles. J Dermatol. (2013) 40:172–6. doi: 10.1111/1346-8138.12046, PMID: 23252484

[20] Kwon SH Choi JY Ahn GY Jang WS Shin JW Na JI . The efficacy and safety of microneedle monopolar radiofrequency for the treatment of periorbital wrinkles. J Dermatol Treat. (2021) 32:460–4. doi: 10.1080/09546634.2019.1662880, PMID: 31500484

[21] Cho S Choi YJ Kang JS. Improvement of periorbital wrinkles treated with an invasive non-insulated microneedle pulsed electric signal device. Med Lasers. (2016) 5:34–8. doi: 10.25289/ML.2016.5.1.34

[22] Yogya Y Wanitphakdeedecha R Wongdama S Nanchaipruek Y Yan C Rakchart S. Efficacy and safety of using noninsulated microneedle radiofrequency alone versus in combination with polynucleotides for treatment of periorbital wrinkles. Dermatol Ther. (2022) 12:1133–45. doi: 10.1007/s13555-022-00729-7, PMID: 35501660 PMC9110589

[23] Wu X Liu Y Zhu J Yu W Lin X. A prospective trial of the microneedle fractional radiofrequency system application in the treatment of infraorbital dark circles. Clin Cosmet Investig Dermatol. (2022) 15:1293–300. doi: 10.2147/CCID.S372409, PMID: 35836477 PMC9275426

[24] Seul TW Park JH Kim JY Ryu HJ. Efficacy assessment of a pulsed-type bipolar radiofrequency microneedling device for treating facial acne vulgaris using a skin-color imaging system: a pilot study. Appl Sci. (2023) 13:2114. doi: 10.3390/app13042114

[25] Ruiz-Esparza J Gomez JB. Nonablative radiofrequency for active acne vulgaris: the use of deep dermal heat in the treatment of moderate to severe active acne vulgaris (thermotherapy): a report of 22 patients. Dermatologic Surg. (2003) 29:333–9. doi: 10.1046/j.1524-4725.2003.29081.x, PMID: 12656809

[26] Liu TM Sun YM Tang ZY Li YH. Microneedle fractional radiofrequency treatment of facial photoageing as assessed in a split-face model. Clin Exp Dermatol. (2019) 44:e96–e102. doi: 10.1111/ced.13924, PMID: 30710383

[27] Hongcharu W Gold M. Expanding the clinical application of fractional radiofrequency treatment: findings on rhytides, hyperpigmentation, rosacea, and acne redness. J Drugs Dermatol. (2015) 14:1298–304. PMID: 26580880

[28] Nilforoushzadeh MA Alavi S Heidari-Kharaji M Hanifnia AR Mahmoudbeyk M Karimi Z . Biometric changes of skin parameters in using of microneedling fractional radiofrequency for skin tightening and rejuvenation facial. Skin Res Technol. (2020) 26:859–66. doi: 10.1111/srt.12887, PMID: 32585051

[29] Peterson JD Palm MD Kiripolsky MG Guiha IC Goldman MP. Evaluation of the effect of fractional laser with radiofrequency and fractionated radiofrequency on the improvement of acne scars. Dermatologic Surg. (2011) 37:1260–7. doi: 10.1111/j.1524-4725.2011.02110.x, PMID: 21834933

